# The Role of Dendritic Cells in the Host Response to Marek’s Disease Virus (MDV) as Shown by Transcriptomic Analysis of Susceptible and Resistant Birds

**DOI:** 10.3390/pathogens11111340

**Published:** 2022-11-13

**Authors:** Pankaj Chakraborty, Richard I. Kuo, Zhou Wu, Katrina M. Morris, Bernadette M. Dutia, Pete Kaiser, Jacqueline Smith

**Affiliations:** 1 The Roslin Institute and R(D)SVS, University of Edinburgh, Easter Bush, Midlothian EH25 9RG, UK; 2 Department of Medicine and Surgery, Faculty of Veterinary Medicine, Chattogram Veterinary and Animal Sciences University, Chattogram 4225, Bangladesh; 3 Roslin Innovation Centre, Wobble Genomics, Easter Bush Campus, Midlothian EH25 9RG, UK

**Keywords:** chicken, Marek’s disease virus, disease resistance, dendritic cells, RNAseq, transcriptome, mir-124a, AP-1

## Abstract

Despite the successful control of highly contagious tumorigenic Marek’s disease (MD) by vaccination, a continuous increase in MD virus (MDV) virulence over recent decades has put emphasis on the development of more MD-resistant chickens. The cell types and genes involved in resistance therefore need to be recognized. The virus is primarily lymphotropic, but research should also focus on innate immunity, as innate immune cells are among the first to encounter MDV. Our previous study on MDV–macrophage interaction revealed significant differences between MHC-congenic lines 6_1_ (MD-resistant) and 7_2_ (MD-susceptible). To investigate the role of dendritic cells (DCs) in MD resistance, bone-marrow-derived DCs from these lines were infected with MDV in vitro. They were then characterized by cell sorting, and the respective transcriptomes analysed by RNA-seq. The differential expression (DE) of genes revealed a strong immune activation in DCs of the susceptible line, although an inherent immune supremacy was shown by the resistant line, including a significant expression of tumour-suppressor miRNA, gga-mir-124a, in line 6_1_ control birds. Enrichment analysis of DE genes revealed high expression of an oncogenic transcription factor, AP-1, in the susceptible line following MDV challenge. This research highlights genes and pathways that may play a role in DCs in determining resistance or susceptibility to MDV infection.

## 1. Introduction

Marek’s disease (MD), caused by *Gallid alphaherpesvirus 2*, is an economically important tumorigenic, lymphotropic viral disease of chickens. The virus is commonly known as Marek’s disease virus (MDV). Although the virus is well-known for its association with lymphoid cells, MDV infection in other cells such as fibroblasts [1], EACs (ellipsoid-associated cells) [2] and phagocytes [3] has also been documented. Despite its oncogenic nature, MD is the first of its kind to be controllable by large-scale global vaccination programs [4,5,6]. However, the virulence of MDV has been increasing in recent decades, as the current MDV vaccines do not induce sterile immunity, but only protect chickens from developing tumours [7,8]. With the continuing evolution of MDV virulence, it is increasingly important to identify the genes responsible for exerting resistance to MD, and to explore the cell types in which this phenotype is expressed. This will allow for the selection and breeding of more resistant chickens.

Among the antigen-presenting cells (APCs), dendritic cells (DCs) efficiently present antigens to T cells, and thus play a crucial role in the initiation of adaptive immune responses. Because MDV infection is strictly cell-associated, T-cell-mediated immunity is likely to play a more significant role than humoral immunity [9]. Research on MDV pathogenesis has largely been focused on T cells due to the lymphotropic nature of MDV, and information regarding the roles of macrophages and DCs in MDV pathogenesis is very limited [10]. Nonetheless, DCs are the least-studied cells regarding MDV infections in chickens, and therefore require further examination. Moreover, there is still a lack of knowledge regarding the type and function of DCs in the initiation of adaptive immune responses such as the presentation of MDV antigen and the priming of T cells by DCs in chickens [10].

Immune cells such as phagocytes as well as B and T cells are infected by MDV during different stages of its life cycle [11], and the resistance signatures can be expressed in any or all of these cells. Among the immune cells, B and T cells can be readily infected with MDV under both in vivo and in vitro conditions. However, it is important to examine MDV–phagocyte interactions, as macrophages/DCs are the innate immune cells with which MDV first interacts. The MDV infection of DCs has not been described in vivo, perhaps due to the difficulty in isolating adequate numbers of DCs for downstream analysis following MDV infection. To overcome the problems of in vivo MDV–DC (or phagocyte) infection studies, an in vitro infection model was developed [3] and chicken bone-marrow-derived DCs (BMDCs) were generated and characterized from outbred chickens using a previously described protocol [12]. Using this model in an earlier experiment, we infected bone-marrow-derived macrophages (BMDMs) from MD-resistant (line 6_1_) and MD-susceptible (line 7_2_) chickens and conducted a transcriptomic analysis [13]. These two inbred chicken lines are exceptional in the sense that, despite sharing the same MHC haplotype, they exhibit very different phenotypes with regard to MDV resistance [14,15]. This highlights the role of non-MHC genetics in this disease resistance. To complement and add to this previous study, BMDCs from lines 6_1_ and 7_2_ were infected with MDV-infected CEFs (chicken embryo fibroblasts) and characterized by FACS (fluorescence-activated cell-sorting) and RNA-seq. Transcriptomic analysis was carried out with the aim of exploring the role of DCs pre- and post-MDV infection in vitro.

## 2. Materials and Methods

### 2.1. Experimental Birds 

Specific-pathogen-free (SPF) chickens from inbred research lines 6_1_ and 7_2_ [16] were used in this study. Line 6_1_ birds were bred in the Poultry Production Unit at the Institute for Animal Health, Compton, UK and reared in the poultry unit of The Moredun Research Institute, Edinburgh, UK, while line 7_2_ chickens were bred and reared at the National Avian Research Facility (NARF), Edinburgh, UK. For chicken embryos, chicken layer line J was used (an intercross from 9 lines, originally bred from Brown Leghorn chickens at the Poultry Research Centre, Edinburgh). They were bred and conventionally raised at the NARF.

### 2.2. Marek’s Disease Virus

The virus used in this study was CVI988 *UL41* eGFP and it was generated from a BAC construct of vaccine strain CVI988 (Rispens) of MDV serotype 1, in which the *UL41* gene was replaced with eGFP (enhanced green fluorescent protein) under the control of the murine phosphoglycerol kinase promoter [17]. The presence of eGFP indicates MDV replication. MDV replication is independent of *UL41* deletion, as mutant strains replicate as well as the parental strain in culture [18].

### 2.3. Cell Cultures

Initially 12–15 chicken embryos of 9–11 days old were used to collect chicken embryo fibroblasts (CEFs). The CEFs were cultured in T_175_ flasks as described previously [13]. The MDV-BAC virus was initially grown and propagated in CEF cultures [19]. Isolation of chicken bone marrow cells was carried out from 3–6-week-old birds and BMDCs were cultured as per the standard method [12]. In order to obtain approximately 1 × 10^7^ BMDCs at harvest on day 4 of culture, bone marrow cells were seeded at a concentration of approximately 1.2 × 10^6^ cells/mL. Cells were cultured in T_75_ flasks at 41 °C with 5% CO_2_ using RPMI-1640 medium supplemented with 10% heat-inactivated chicken serum (CS), 1% L-glutamine and 0.1% pen-strep. Recombinant chicken interleukin-4 (chIL-4) and granulocyte-macrophage colony stimulating factor (chCSF-2 or GM-CSF) [12] were added to the BMDC culture and medium was refreshed every 2 days.

### 2.4. Co-Culture Infection Experiments and FACS (Fluorescence-Activated Cell Sorting)

Virus-infected CEFs were used to infect BMDCs because MDV is strictly cell-associated in culture. Co-culture infection experiments and FACS were performed as described previously [13] with the exception that the BMDCs were infected with sorted MDV-infected CEFs on day 4 of culture at an infection ratio of 1:2.5 (CEF:BMDC) in RPMI-1640 medium containing 10% FBS (Gibco), 1% pen-strep and 1% L-glutamine. Co-cultured cells were incubated at 41 °C with 5% CO_2_ for 1 day, and then harvested for downstream experiments.

### 2.5. Cell and Sample Preparation for RNA-Sequencing (RNA-seq)

Control and infected BMDCs from inbred chicken lines 6_1_ and 7_2_ were sorted at 1 dpi based on eGFP and CD45 expression as described previously [13]. RNA was extracted using RNeasy Mini Kits (Qiagen, Manchester, UK), and DNase treatment of RNAs was carried out with Ambion Turbo DNA-free kits (Life Technologies, Paisley, UK). RNA quantification was performed using Qubit RNA Assay kit with the Qubit 2.0 Fluorometer (Invitrogen, Inchinnan, UK). Three separate biological replicates for each of the control and infected BMDCs were used for RNA preparation and subsequent sequencing.

### 2.6. RNA-seq and Analysis

RNA sequencing was performed by the Edinburgh Genomics sequencing facility (Edinburgh, UK) using a pool of individually barcoded RNA samples representing 3 biological replicates for each of the control and infected BMDCs. Paired-end sequencing (125 bp reads) was carried out on an Illumina HiSeq 2500 platform using an Illumina TruSeq Rapid SBS Kit (Illumina, Little Chesterford, UK). The quality of the RNA-seq reads was evaluated using the software FastQC [20]. Adapter sequences (Illumina TruSeq v3 adapters) were trimmed from the FASTQ sequences using Cutadapt software (v1.6) [21] with a minimum length cut-off at 50 bp. Trimmed reads were analysed to explore the differentially expressed (DE) genes in MDV-infected and control BMDCs. The data analysis pipeline included the following steps: The short reads were aligned to the chicken reference genome sequence (Galgal4, Ensembl release 78) using Bowtie and TopHat software packages [22]. Following alignment of the RNA-seq reads, HTSeq-count was used to calculate counts per million (CPM) for each gene [23]. The differential gene expression was then analysed using edgeR (empirical analysis of digital gene expression in R) software [24]. Differentially expressed genes (DEGs) were filtered using an FDR (false discovery rate) of 0.05 and fold change (FC) > 2. Viral transcript expression was measured by mapping reads to the CVI988 MDV genome (Accession: DQ530348) using STAR [25] with default options, except we scaled down --enomeSAindexNbases to 7, as suggested, due to the small size of the viral genome. Mapped reads were counted using featurecounts [26]. The mapping statistics was summarized to assess the number of reads mapped to the viral gene. Expression is detailed as transcripts per million (TPMs). Differential expression comparisons were performed using DESeq2 [27].

### 2.7. In Silico Functional Analysis of DE Genes

Ingenuity Pathway Analysis (IPA) software (Qiagen—https://digitalinsights.qiagen.com/products-overview/discovery-insights-portfolio/analysis-and-visualization/qiagen-ipa/ (accessed on 27 September 2021)) was used to reveal biological pathways and functions pertaining to the DEGs identified in our analyses. P-values were calculated using the right-tailed Fisher Exact Test (threshold *p* < 0.05). Enriched pathways, gene ontologies, miRNA targets and transcription factor binding sites were identified using WebGestalt (WEB-based GEne SeT AnaLysis Toolkit) algorithms (http://www.webgestalt.org/ (accessed on 28 September 2021)) [28]. Over-representation analysis (ORA) was carried out using the network functional database to identify both transcription factor and miRNA targets. The ‘chicken genome’ was used as the background reference. Results showing an FDR < 0.05 were considered significant. gProfiler (https://biit.cs.ut.ee/gprofiler/gost (accessed on 5 October 2021)) [29] provided functional analysis of the host responses in each line. DEGs were examined as an ordered list and queried against the *Gallus gallus* background reference.

### 2.8. Determination of Potential MDV QTL Candidate Genes

The BioMart data mining tool within the Ensembl database (release 78) (http://www.ensembl.org/index.html) was used to identify differentially expressed genes located in genomic regions previously identified as being under QTLs for MDV resistance [30,31] (Supplementary File S1).

## 3. Results

### 3.1. BMDC Infection in Resistant and Susceptible Lines

**Figure 1:**
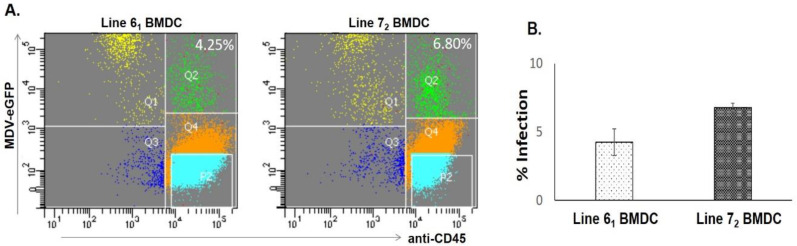
(**A**) The in vitro infection of DCs from two inbred lines with Marek’s disease virus (MDV). Chicken bone-marrow-derived DCs (BMDCs) from lines 6_1_ and 7_2_ were cultured with chIL-4 and colony stimulating factor 2 (chCSF-2) for 4 days. On the day of infection, pre-sorted eGFP+CD45- chicken embryo fibroblasts (CEFs) were co-cultured with BMDCs at a ratio of 1:2.5 (CEF:BMDC). At 1 dpi, in vitro-infected BMDCs were characterized by FACS for the expression of eGFP and CD45. Data shown are the average percentage of infection of four independent experiments. Distribution of cells: Q1, infected CEFs; Q2, infected BMDCs; Q3, uninfected CEFs; Q4, uninfected BMDCs. (**B**) Graph showing means and respective standard errors of the mean (SEM) of the percentages of infected cells found within the four biological replicates from lines 6_1_ and 7_2_ BMDCs during cell-sorting experiments.

Pre-sorted MDV-infected CD45-eGFP+CEFs were co-cultured with chicken BMDCs from the two inbred lines (6_1_ and 7_2_), and analysis of BMDCs expressing CD45 and eGFP during co-culture cell sorting experiments on 1 dpi revealed that the average proportion of infected DCs was 1.6% higher in line 7_2_ (MD-susceptible; 6.8%) than in line 6_1_ (MD-resistant; 4.25%) (Figure 1A). The means and respective standard errors of the mean (SEMs) of the percentages of infected cells from four independent experiments are shown in Figure 1B.

### 3.2. Analysis of Gene Expression in BMDCs of Resistant and Susceptible Lines

Analysis of RNA-seq data was carried out to explore differences in gene expression in control and infected BMDCs from the two inbred lines. The infected BMDCs were purified using flow sorting based on the expression of eGFP-MDV and CD45. Comparisons were made as follows: (1) Control BMDCs (line 6_1_) vs. control BMDCs (line 7_2_)—to see which genes were inherently differentially expressed between susceptible and resistant birds. (2) Control BMDCs vs. infected BMDCs for both lines 6_1_ and 7_2_—to define the host response to infection in each line of birds. (3) (Infected BMDCs − control BMDCs) from line 6_1_ vs. (infected BMDCs − control BMDCs) from line 7_2_—to examine differences in host response to MDV infection between susceptible and resistant birds. The numbers of significant DEGs (FDR < 0.05; FC > 2) used for downstream functional analyses are provided in Table 1. The full lists of DEGs in BMDCs from lines 6_1_ and 7_2_ are provided in Supplementary File S2.

### 3.3. Inherent Differences Exist between the Two Inbred Lines

In order to compare basal gene expression in each line, control birds were compared. An almost equal number of genes were differentially expressed in each line prior to infection (477 genes were more highly expressed in line 6_1_ and 478 genes were higher in line 7_2_) (Supplementary File S2). However, a different immune-related gene profile was seen in line 6_1_ (MD-resistant) compared to line 7_2_ (MD-susceptible). Genes more highly expressed in line 6_1_ included interleukins, interleukin ligands, receptors and accessory proteins (*IL8*, *IL8L1*, *IL13RA1*, *IL13RA2*, *IL20RA*, *IL1RAPL2H1*), CD antigen genes (*CD200*, *CD109*, *CD2*), chemokine ligand *CCL4*, tumour necrosis factor (TNF) receptor-associated factors (*TRAF1*, *TRAF2* and *TRAF3*) and TNF superfamily member *TNFSF13B*. Other genes included interferon alpha-inducible proteins (*IFI6* and *IFI27L2*), the interferon regulatory factor *IRF1*, the myxovirus resistance gene (*MX1*), the avidin gene (*AVD*), the lysozyme gene (*LYZ*), inducible nitric oxide synthase (*INOS*) and the basic leucine zipper ATF-like transcription factor 3 gene (*BATF3*). Among the highly expressed DEGs in line 7_2_, genes with known immune functions included some interleukins, chemokine ligands and receptors (*IL12B*, *IL1R2*, *IL15*, *CCL17*, *CXCR4*), a couple of TNF receptor superfamily members (*TNFRSF18*, *TNFRSF25H*), Toll-like receptor gene *TLR7*, the beta defensins *AvBD1* and *AvBD2* and other CD antigen genes (*CD200R1L*, *CD300L-S1*, *CD300LG*, *CD14*, *CD3D*, *CD3E*, *CD1A1, CD247*, *CD83*, *CD244*, *CD80*). Hence, there were inherent differences between the two lines with regard to immune gene expression.

### 3.4. Host Response to MDV Infection in Susceptible Birds

In the MD-susceptible line (7_2_), 843 genes were upregulated and 678 genes were downregulated compared to control during the host response against MD (Supplementary File S2). Downregulated immune genes were low in number, consisting of only a few interleukins and chemokines (*IL15*, *IL1R2*, *IL1RAPL2HI*, *CCL17*, *CXCR4*); a couple of TLRs (*TLR4*, *TLR7*); one TNF gene (*TRAF3IP3*); some interferon regulatory genes (*IRF2BP2*, *IFNGR2*, *SOCS2*) and *CD79B*. Contrarily, many immune-related genes were upregulated, including several interleukins and chemokines (*IL6*, *IL16*, *IL23R*, *IL2RA*, *IL13RA2*, *IL18RAP*, *CCL19*, *CCL20*), *TLR3*, TNFs (*TNFAIP2*, *TNFAIP6*, *TNFSF10*, *TNFRSF8*, *TNFRSF10B*, *TNFRSF11B*, *TRAF1*, *TRAF3*, *TRAF3IP2*), genes involved in controlling the interferon response (*IFIT5*, *IFITM3*, *IFITM5*, *IRF1*, *IRF10*, *IFI6*, *IFI27L2*, *IFNW1*, *STAT1*, *STAT2*, *SOCS7*) and some other immune-related genes (*INOS*, *GZMA*, *MX1*, *NFκBIZ*). This indicates a strong immune response against MDV in the susceptible birds.

### 3.5. Host Response to MDV Infection in Resistant Birds

After MDV challenge, 760 genes were upregulated and 722 genes were downregulated in line 6_1_ (MD-resistant) (Supplementary File S2). Among them, a few immune-related genes were highly expressed, whereas the expression of several immune genes decreased. Immune genes with lower expression included interleukins and their receptors (*IL1B, IL8, IL1RL1, IL10R2, IL20RA* and *IL1RAPL2H1*), chemokines and their receptors (*CCR2, CCR7, CCL4, CCL17, CCL26, CXCL1, CXCL14*), Toll-like receptors (*TLR1A, TLR1B, TLR15*), some TNF genes (*TNFSF13B, TNFRSF6B, TNFRSF11A, TRAF3IP3*), interferon regulatory genes (*IRF5, IRF8, IRF2BP2, IRF2BPL, IFNGR2*), the suppressor of cytokine signalling genes (*SOCS1, SOCS2, SOCS3*) and some other immune-related genes, such as *AVD, LYZ, AMIGO2, CD79B, CHIR-B2* and *TGFB1*. In contrast, only a few immune-related genes were upregulated in this line during the response to MDV. These included a couple of TNF superfamily members (*TNFSF15, TNFRSF108*), some interferon regulatory genes (*IRF4, IFIT5, IFNW1, IFI6, SOCS7*) and the *IL23R* interleukin receptor. This suggests that the more resistant birds do not need to mount as robust an immune response to MDV as their more susceptible counterparts. Indeed, the suppression of such genes would prevent an inappropriate and excessive inflammatory response, which is often the main reason for the pathology seen in the host.

### 3.6. Comparison of Host Response to Infection between the Two Lines

There were differences in gene expression levels between BMDCs from the two lines after infection (317 DEGs were highly expressed in line 6_1_ and 668 DEGs were expressed at a higher level in line 7_2_) (Supplementary File S2). In the resistant line (6_1_), the upregulated immune genes were few in number, and included *ILIRAPL2H1*, a couple of TNFs (*TNFSF11, TNFSF15*), chicken immunoglobulin-like receptors and adhesion molecule (*CHIR-B2, CHIR-IG1-5, CHIR-AB-600, AMIGO2*).

On the other hand, genes more highly expressed in the susceptible line (7_2_) included several interleukins and interleukin receptors (*IL1β, IL6, IL1RL1, IL22RA1, IL17RE, IL17RC*); chemokines (*CCL1, CCL17, CCL26, CX3CL1, CXCL12, CXCL14*); TLRs (*TLR2H, TLR21*); a number of TNFs (*TNFAIP2, TNFAIP6, TNFSF10, TNFRSF6B, TNFRSF11B, TNFRSF25H, TRAF3IP3*); various interferon controlling genes (*IFI6, IRF7, IFITM5, SOCS1, SOCS2, SOCS3*); a few other immune-related genes (*JAK3, GZMA, INOS*); B-cell linker gene (*BLNK*); and transforming growth factor beta genes (*TGFB1* and *TGFB2*).

**Figure 2:**
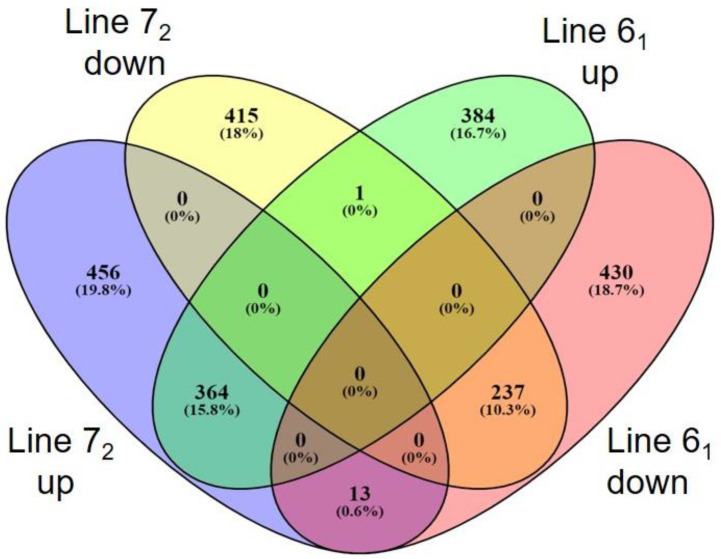
Venn diagram showing the overlap of differentially expressed genes between lines 6_1_ and 7_2_ after infection by MDV. Numbers of genes up- and downregulated are indicated.

Hence, disparity in the upregulation of immune genes was identified between resistant and susceptible lines following MDV infection. The distinctive nature of each response can be visualized in the Venn diagram shown in Figure 2. In line 7_2_, 456 genes were uniquely upregulated and 415 down, while in line 6_1_, 384 genes were upregulated only in this line and 430 were uniquely downregulated. Thirteen genes (*ACSL6, ADAM12, ADAMTS15, ADAMTSL1, CD72AG, COL6A3, CSPG4, FSCN1, PTGS2, LOC101749645, PRDM8, TNFAIP6* and *TNIP3*) saw their expression increase in line 7_2_ while being downregulated in line 6_1_. Only 1 gene (*GNAI1*) was seen to show increased expression in line 6_1_ while showing reduced expression in line 7_2_.

### 3.7. Expression Profiles of MicroRNAs

MicroRNAs (miRNAs) are small, non-coding RNAs that regulate gene expression by directing them for translational repression or degradation [32]. Although samples were not specifically sequenced to capture small RNAs, some miRNAs have been identified as being differentially expressed in our study. Looking at inherent gene expression in control birds, we see that gga-mir-1797 was more highly expressed in line 7_2_, whereas gga-mir-22 expression was higher in line 6_1_. Upon infection, gga-mir-3064 and gga-mir-6690 were downregulated in line 6_1_. In line 7_2_, gga-mir-3523 was upregulated in response to virus while expression of gga-mir-1618, gga-mir-3064 and gga-mir-34a was decreased. Comparison of expression between the lines after infection demonstrates that gga-mir-3064 and gga-mir-6690 were more highly expressed in susceptible birds (7_2_), and gga-mir-22 showed greater expression with the more resistant phenotype (6_1_). Although the function of these miRNAs is largely unknown, it is interesting to note that mir-22, which has been associated with tumour-suppressor activity [33], was more highly expressed in line 6_1_ while expression of another tumour-suppressor, gga-mir-34a, was decreased in line 7_2_.

### 3.8. Viral Gene Expression

Viral gene expression after infection in lines 6_1_ and 7_2_ was determined, and is presented as transcripts per million reads in Supplementary File S3. The small number of reads seen in control samples were background. A high level of viral expression was seen in the infected samples. Differences in the expression of the viral transcripts in each of the two lines are shown in Supplementary File S4. The expression of viral transcripts varied between the two lines, with some transcripts found to be present at up to four times higher levels in line 7_2_ as compared to line 6_1_, although some transcripts were more highly expressed in line 6_1_ (with regard to TPM values). The average TPM value was 1.1 for line 7_2_/line 6_1_, which reflects the cell-sorting results. It would therefore appear that similar numbers of dendritic cells became infected in the two lines, and there were also similar levels of viral gene expression. This would indicate that the difference in host response to MDV infection in dendritic cells is due to how the cells respond to infection and not to differences in viral infection in the cell.

### 3.9. Downstream Analysis of Differentially Expressed Genes

**Figure 3:**
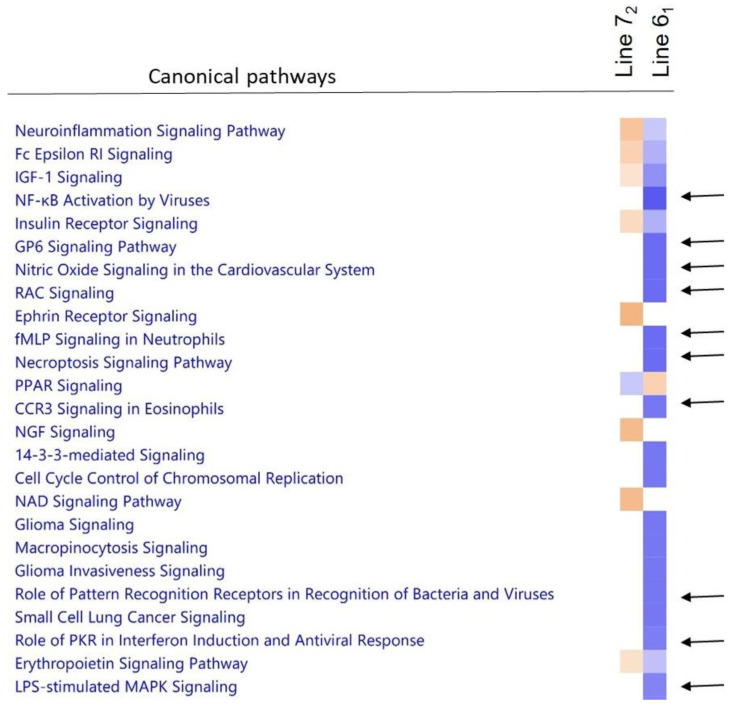
Comparison of canonical pathways represented by genes expressed in BMDCs at inherently different levels in the two lines studied. Orange colour denotes upregulation in the MD-susceptible line (7_2_) and blue colour represents upregulation in the MD-resistant line (6_1_). Arrows indicate some of the pathways showing notable basal differential gene expression in each line.

Inherent differences in biological pathways were observed when comparing gene expression in BMDCs of uninfected control birds between the two lines. Genes involved in pathways such as those for NFĸB activation, signalling of GP6, nitric oxide, RAC, fMLP, necroptosis, CCR3, the role of pattern recognition receptors, interferon induction and MAPK signalling were expressed at a higher level in line 6_1_ compared to line 7_2_ (Figure 3). Thus, even before infection, it can be seen that the more resistant line was primed to attack the MDV pathogen in a way that the more susceptible line was not. Analysis of genes more highly expressed in line 6_1_ showed an enrichment of genes that are targets for mir124a (Supplementary File S5). This microRNA is known to act as a tumour suppressor [34], and to be particularly associated with normal neuronal development and function [35].

**Figure 4:**
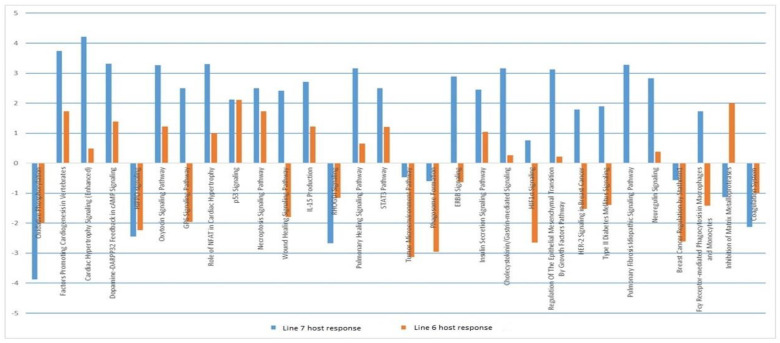
A comparison of pathways activated/inhibited in lines 6_1_ and 7_2_ in response to MDV challenge.

The biological pathways involved during the host response in each line were examined. Figure 4 compares pathways which were activated or inhibited in host dendritic cells after infection with MDV. Several pathways were seen to be more downregulated in line 6_1_ compared with line 7_2_. These included GP6 signalling, tumour microenvironment, ERBB signalling, HIF1A signalling, HR2 signalling and phagocytosis. These are all processes associated with the immune system and/or tumour progression, and were more highly activated in the susceptible line 7_2_ birds after challenge with MDV.

Using the WebGestalt tool to examine DEGs for specific enrichment showed an over-representation of genes associated with particular transcription factors and as targets for specific miRNAs. A significant number of genes upregulated in the resistant line 6_1_ birds after infection contain binding sites for transcription factors, including CREBP1, which can act as a tumour suppressor [36], and RFX1, which is a regulator of MHC Class II genes [37]. Many of these DEGs are also targets for the miRNA mir-153 (amongst others), which is known to act as a tumour suppressor and reduce oncogenic processes. This is in contrast to DEGs in line 7_2_ that are over-represented by genes that have binding sites for transcription factors including LEF1, AFP1 and p53—all of which have been associated with cancer progression [38,39,40]. We also observed an enrichment of genes acting as targets for miRNA mir-374 (amongst others), which is known to be associated with lymphoma [41]. A complete list of enriched factors found in DEGs either up- or downregulated in each of the lines can be found in Supplementary File S6. When a direct comparison of lines was made, we observed that DEGs in line 7_2_ showed an over-representation of genes with binding sites for AP-1 (Table 2). In order to examine genes upregulated in each line more closely, the gProfiler tool was used. Significant GO terms, pathways and transcription factor binding sites for DEGs in each line are shown in Supplementary File S7.

### 3.10. Identification of Putative Candidate Genes for Resistance/Susceptibility to MD

To explore potential candidate genes for resistance or susceptibility to MD, DEGs were analysed between the two lines pre- and post-MDV infection by mapping them to regions underlying known QTLs (Supplementary File S1). Genes significantly differentially expressed and lying within defined QTL regions can be highlighted as strong candidates for having a role in resistance/susceptibility to MDV. Candidates showing differential inherent expression between the two lines included *FGFR3* and *SLBP*, which were more highly expressed in line 6_1_, and *ST6GALNAC4*, which showed higher expression in line 7_2_. Examination of DEGs following MDV challenge indicated *CERCAM*, *ST3GAL1, FGFRL1* and *TMEM71* as potential candidates for increased susceptibility to MDV infection. The above-mentioned genes lie under QTLs reported previously [30,31]; however, all genes listed in Supplementary File S2 that are associated with a known QTLR can be considered potential candidates for resistance/susceptibility to MDV.

## 4. Discussion

To investigate the role of dendritic cells in the host response to MDV, BMDCs from MD-resistant (6_1_) and MD-susceptible (7_2_) chicken lines were infected with MDV-infected CEFs using a pre-established in vitro infection model and accordingly characterized on 1 dpi by FACS and RNA-seq analysis. Despite sharing the same MHC haplotype, these two inbred chicken lines express opposite phenotypes following infection with MDV, as shown by the mortality rates, ranging from 7% to 94% in resistant and susceptible chickens, respectively [42]. A high difference in the percentage of infection was also observed between the two lines when BMDMs (bone-marrow-derived macrophages) from these two lines were infected in vitro with MDV, revealing that line 7_2_ BMDMs were three times more susceptible than those of line 6_1_ [13]. As phagocytes, BMDCs from these two lines could be assumed to be infected by MDV at levels similar to BMDMs. However, the percentage of BMDCs infected in this study was only around 1.6% higher in the susceptible line compared to the resistant line, despite using a high ratio of infection. In this context it is worth mentioning that in order to obtain a sufficient number of infected BMDCs during cell sorting, a higher infection ratio (CEF:BMDC; 1:2.5) was used here than in our previous study (CEF:BMDM; 1:5) [13].

A possible interpretation of low infection in DCs could be that while BMDMs were cultured in the presence of chicken CSF-1, BMDCs were cultured with chIL-4 and chCSF-2, leading to differential transcription profiles in these two cell types, which might affect their susceptibility to infection [3]. Another potential reason for lower infection in DCs may be related to their function. While macrophages are well-equipped for terminating invading microorganisms, DCs are engaged in the initiation of adaptive immunity by the efficient processing and presentation of antigens to T cells [43]. Therefore, macrophages probably phagocytose more pathogens to destroy them at a greater rate, and DCs internalize a lesser amount of virus sufficient for processing and presenting antigens to T cells. There is also evidence that phagosomal degradation and acidification are much lower in DCs compared to macrophages [44]. These factors might have been the cause of the lower MDV infection rate seen in the current study. However, further studies are needed to clarify this.

Transcriptomic analysis of MDV-infected DCs has not previously been carried out, either in vivo or in vitro. To illustrate the role of DCs during MDV infection in vitro, DEGs of control and infected BMDCs from resistant (6_1_) and susceptible lines (7_2_) were analysed and compared. Although infection rates of susceptible and resistant lines were comparable, the inherent levels of immune gene expression and subsequent changes after challenge were very different. Differences in inherent gene expression between the lines may account for some of the differential resistance seen in these birds, and may help to explain the lower morbidity in line 6_1_ following infection. It was observed that, after MDV infection, many immune genes were downregulated in line 6_1_ BMDCs while their upregulated expression was seen in line 7_2_. This finding is in agreement with our previous study in BMDMs [13], and it also supports the concept of MDV latency in resistant chickens (6_1_) as not being recognized by the host immune responses [45], presumably by the lower expression of immune genes.

Differing gene expression profiles were seen in response to MDV challenge depending on which line was being examined. A stronger immune system activation was seen in the more susceptible line 7_2_ birds, although the more resistant line 6_1_ birds appeared to be more inherently immune-primed. Biological pathways significantly activated/repressed were those involved in the immune system, those related to neuronal pathways and those involved with oncogenesis, which is what might be expected considering the profile of MDV infection.

Upon MDV challenge, we saw unique responses in each of the two lines, with some genes showing completely contrasting expression. Genes showing increased expression in line 7_2_ but downregulation in line 6_1_ included *ACSL6, ADAM12, ADAMTS15, ADAMTSL1, CD72AG, COL6A3, CSPG4, FSCN1, PTGS2, LOC101749645, PRDM8, TNFAIP6* and *TNIP3*. Some of these genes have functions that are obviously relevant in MDV pathogenesis. These include genes with a role in neuronal processes (*ACSL6, ADAM12, CSPG4, PRDM8*) and the immune system (*CD72AG, PTGS2, TNIP3*). Several other genes have roles in the cytoskeleton/extra-cellular matrix (*ADAMTSL1, COL6A3, FSCN1, TNFAIP6*). Extracellular matrix degradation is one of the consequences of MDV infection [46]. There was only 1 gene (*GNAI1*) that showed increased expression in line 6_1_ but downregulation in line 7_2_. *GNAI1* is a guanine nucleotide-binding protein, and mutations in this gene are known to cause neurodevelopmental disorders [47].

Specific profiling of small RNAs was not carried out, but a few miRNAs were still highlighted as being differentially expressed between the two lines examined in this study. Gga-mir-22 and gga-mir-1797 showed different inherent levels of expression in control birds, while miRNAs gga-mir-22, gga-mir-3064 and gga-mir-6690 responded differently in each line after infection. Very little is known regarding the function of most chicken miRNAs, but they are understood to play a significant role in the regulation of cancer progression. Many of these transcripts have functions as either oncogenes or as tumour suppressors. Indeed, some microRNAs can act in both roles, depending on the physiological environment [48]. With the oncogenic nature of MDV [8,49], it is clear why the role of these molecules may be important. Interestingly, gga-mir-22 was highlighted in line 6_1_ infected DCs this study. Mir-22 is known to be able to function as a tumour suppressor [50], and to have neuroprotective function [51]. MDV affects the nervous system of infected chickens [52,53] and thus higher expression of gga-mir-22 in line 6_1_ may have a protective role against some of the symptoms associated with MDV infection.

When differentially expressed genes in DCs were examined as enriched targets for particular miRNAs, gga-mir-124a was identified as significant in control birds. Basal levels of expression were higher in line 6_1_ for genes that are targets for this miRNA. miR-124 exerts a crucial role in the development of the immune system, regulation of immune responses, and inflammatory disorders [54]. miR-124 has been shown to be critically involved in neurogenesis, migration, morphogenesis and neuronal cell death [55]. It is also known to act as a tumour suppressor [34].

Enrichment analysis of differentially expressed genes also highlighted the fact that genes that were more highly expressed in susceptible line 7_2_ in response to MDV challenge contained a high proportion with binding sites for the activator protein 1 (AP-1) transcription factor. AP-1 mediates gene regulation in response to many different physiological and pathogenic stimuli, including viral infections as well as oncogenic stimuli [56]. With its known role as a proto-oncogene [57,58], it could be hypothesized that, even at the early stages of MDV infection, the virus stimulates the expression of genes which can be regulated by AP-1, thus enabling the downstream transformation of cells later in infection. Expression of the gene encoding the JUN subunit of AP-1 was also seen to be downregulated in the more resistant line 6_1_ birds.

Differentially expressed genes located under known MDV QTL regions were mapped to reveal putative candidate genes for MDV resistance/susceptibility. Genes found to be differentially expressed in this study and found to underlie QTL from two previous independent studies [30,31] were of particular interest. The *SLBP* gene was more highly expressed in line 6_1_ control birds, while *ST6GALNAC4* showed higher expression in line 7_2_. *SLBP* is an RNA-binding protein involved in histone processing and expression [59], while *ST6GALNAC4* is known to be overexpressed in metastases [60]. Following MDV challenge, *CERCAM*, *ST3GAL1, FGFRL1* and *TMEM71* were indicated as potential candidates for increased susceptibility to MDV infection. *CERCAM* encodes a cell adhesion protein involved in leukocyte transmigration across the blood–brain barrier [61]. *ST3GAL1* has been implicated in tumour progression [62] and *TMEM71* has been shown to be upregulated in gliomas [63]. A candidate for increased resistance to MDV is the *FGFR3* gene, which had higher expression in line 6_1_ after MDV challenge. Due to frequent mutations in certain cancers, *FGFR3* is considered an oncogene [64]. However, in tumours of epithelial origin, *FGFR3* can limit tumour growth and thus be considered a tumour suppressor [65].

## 5. Conclusions

Here, for the first time, we studied the role of dendritic cells in response to MDV challenge in vitro and outlined a major role in the host response during the early stage of MDV infection. Although we noted similar levels of infection of BMDC in lines of differing resistance, the underlying gene expression profiles were very different. This was seen at both the basal level in uninfected birds and during the host response to MDV infection in each line. The role of different aspects of the immune response were highlighted, along with the potential involvement of various miRNAs. Several candidate genes for MDV resistance/susceptibility were identified as strong targets for future functional validation.

## Figures and Tables

**Table 1:** The numbers of differentially expressed genes (DEGs) (*p* < 0.05) used for functional analysis.

Category	Genes More Highly Expressed in BMDCs of Line 6_1_	Genes More Highly Expressed in BMDCs of Line 7_2_
Host response in line 7_2_ compared to controls	/	843 ↑ and 678 ↓
Host response in line 6_1_ compared to controls	760 ↑ and 722 ↓	/
Inherent difference between lines	477	478
Difference between lines upon infection	317	668

↑—up-regulated; ↓—down-regulated.

**Table 2:** DEGs upregulated in line 7_2_ after challenge with MDV were enriched with binding sites for AP-1.

Gene Set	Size	Expect	Ratio	*p*-Value	FDR
TGANTCA_AP1_C	1590	38.364	1.6682	0.00002	0.009
AP1_C	409	9.8685	2.432	0.00005	0.009
GGGTGGRR_PAX4_03	1853	44.71	1.5656	0.00006	0.009
AP1_Q4_01	412	9.9409	2.4143	0.00006	0.009
CTTTGA_LEF1_Q2	1683	40.608	1.576	0.00011	0.012
SRY_01	324	7.8176	2.5583	0.00012	0.012
AP1_Q2	380	9.1688	2.3994	0.00013	0.012
CTTTAAR_UNKNOWN	1408	33.973	1.6189	0.00019	0.013
STAT4_01	389	9.386	2.3439	0.00019	0.013
CAGGTA_AREB6_01	1162	28.037	1.6763	0.00027	0.017

## Data Availability

Data have been submitted to European Nucleotide Archive under accession number PRJEB55811.

## Supplementary Materials

The following supporting information can be downloaded at: https://www.mdpi.com/article/10.3390/pathogens11111340/s1, Supplementary File S1: QTL regions for MDV resistance examined in this study; Supplementary File S2: All differentially expressed genes identified by the different comparisons described; Supplementary File S3: MDV transcript counts in each sample (TPM); Supplementary File S4: Differential expression of viral transcripts; Supplementary File S5: Genes that are targets for mir124A are enriched within genes more highly expressed in line 6_1_ control birds, compared to line 7_2_ controls; Supplementary File S6: Enrichment analysis results of DEGs in each line as determined by WebGestalt; Supplementary File S7: gProfiler analysis of DEGs from each line.
