# Supplementary material for: The Role of Dendritic Cells in the Host Response to Marek’s Disease Virus (MDV) as Shown by Transcriptomic Analysis of Susceptible and Resistant Birds
Source: Pathogens. 2022 Nov 13;11(11):1340. doi: 10.3390/pathogens11111340 (PMC9698451; doi:10.3390/pathogens11111340)
Supplement: Supplementary file 1 [file pathogens-11-01340-s001.zip › Supp File S7_grofiler.pptx]

## Slide 1
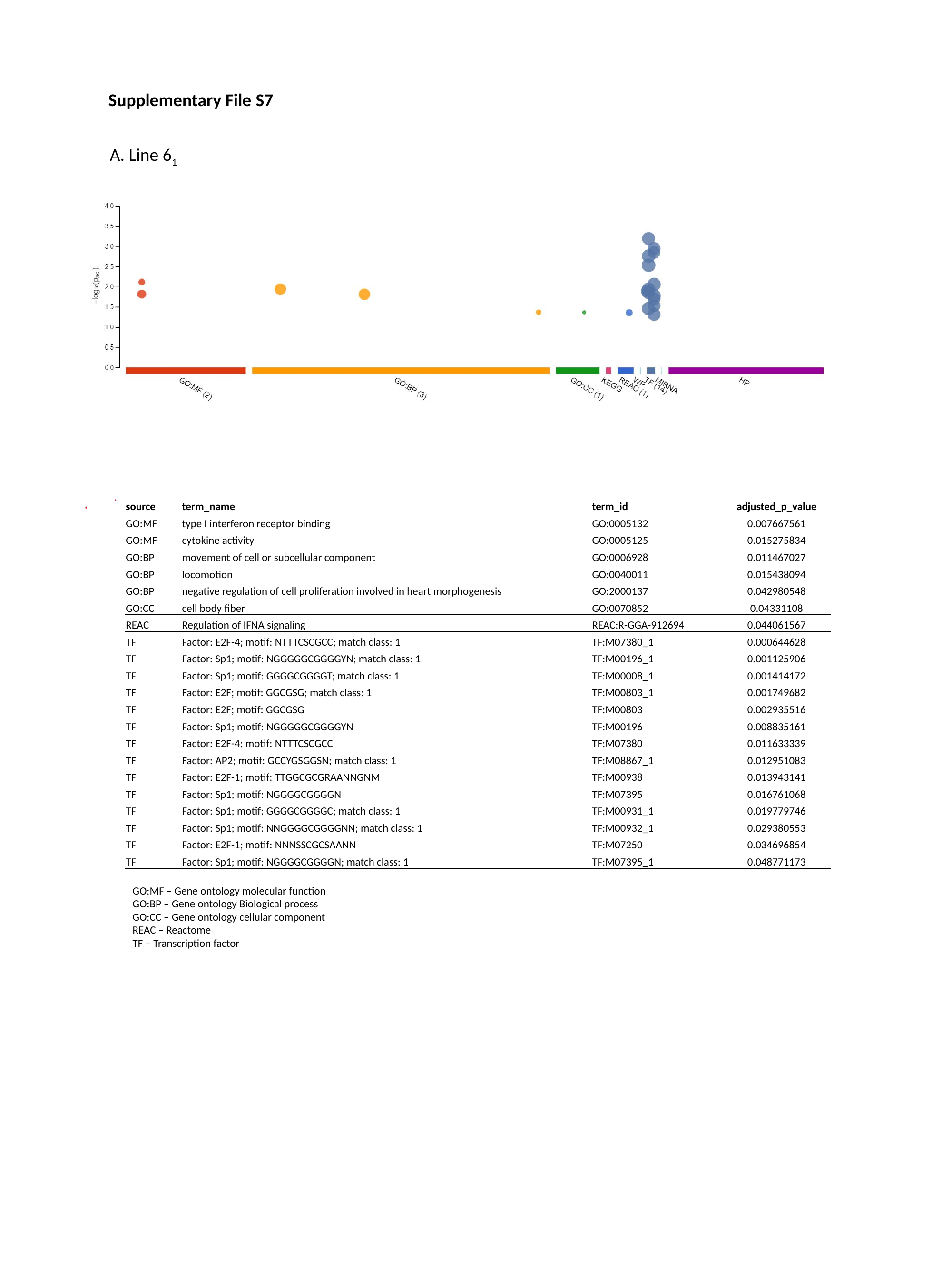

Supplementary File S7
A. Line 61
| source | term\_name | term\_id | adjusted\_p\_value |
| --- | --- | --- | --- |
| GO:MF | type I interferon receptor binding | GO:0005132 | 0.007667561 |
| GO:MF | cytokine activity | GO:0005125 | 0.015275834 |
| GO:BP | movement of cell or subcellular component | GO:0006928 | 0.011467027 |
| GO:BP | locomotion | GO:0040011 | 0.015438094 |
| GO:BP | negative regulation of cell proliferation involved in heart morphogenesis | GO:2000137 | 0.042980548 |
| GO:CC | cell body fiber | GO:0070852 | 0.04331108 |
| REAC | Regulation of IFNA signaling | REAC:R-GGA-912694 | 0.044061567 |
| TF | Factor: E2F-4; motif: NTTTCSCGCC; match class: 1 | TF:M07380\_1 | 0.000644628 |
| TF | Factor: Sp1; motif: NGGGGGCGGGGYN; match class: 1 | TF:M00196\_1 | 0.001125906 |
| TF | Factor: Sp1; motif: GGGGCGGGGT; match class: 1 | TF:M00008\_1 | 0.001414172 |
| TF | Factor: E2F; motif: GGCGSG; match class: 1 | TF:M00803\_1 | 0.001749682 |
| TF | Factor: E2F; motif: GGCGSG | TF:M00803 | 0.002935516 |
| TF | Factor: Sp1; motif: NGGGGGCGGGGYN | TF:M00196 | 0.008835161 |
| TF | Factor: E2F-4; motif: NTTTCSCGCC | TF:M07380 | 0.011633339 |
| TF | Factor: AP2; motif: GCCYGSGGSN; match class: 1 | TF:M08867\_1 | 0.012951083 |
| TF | Factor: E2F-1; motif: TTGGCGCGRAANNGNM | TF:M00938 | 0.013943141 |
| TF | Factor: Sp1; motif: NGGGGCGGGGN | TF:M07395 | 0.016761068 |
| TF | Factor: Sp1; motif: GGGGCGGGGC; match class: 1 | TF:M00931\_1 | 0.019779746 |
| TF | Factor: Sp1; motif: NNGGGGCGGGGNN; match class: 1 | TF:M00932\_1 | 0.029380553 |
| TF | Factor: E2F-1; motif: NNNSSCGCSAANN | TF:M07250 | 0.034696854 |
| TF | Factor: Sp1; motif: NGGGGCGGGGN; match class: 1 | TF:M07395\_1 | 0.048771173 |
GO:MF – Gene ontology molecular function
GO:BP – Gene ontology Biological process
GO:CC – Gene ontology cellular component
REAC – Reactome
TF – Transcription factor

## Slide 2
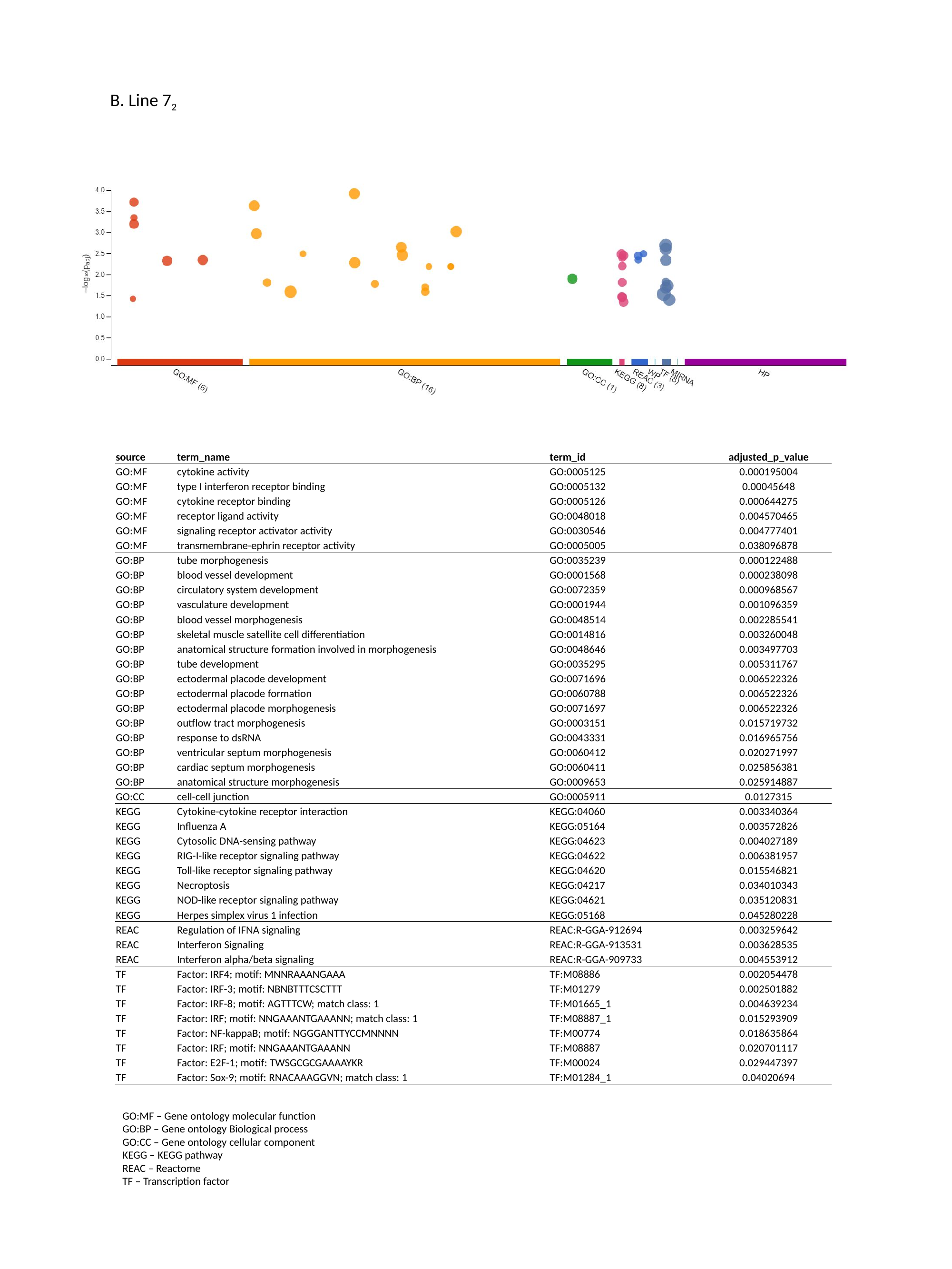

B. Line 72
| source | term\_name | term\_id | adjusted\_p\_value |
| --- | --- | --- | --- |
| GO:MF | cytokine activity | GO:0005125 | 0.000195004 |
| GO:MF | type I interferon receptor binding | GO:0005132 | 0.00045648 |
| GO:MF | cytokine receptor binding | GO:0005126 | 0.000644275 |
| GO:MF | receptor ligand activity | GO:0048018 | 0.004570465 |
| GO:MF | signaling receptor activator activity | GO:0030546 | 0.004777401 |
| GO:MF | transmembrane-ephrin receptor activity | GO:0005005 | 0.038096878 |
| GO:BP | tube morphogenesis | GO:0035239 | 0.000122488 |
| GO:BP | blood vessel development | GO:0001568 | 0.000238098 |
| GO:BP | circulatory system development | GO:0072359 | 0.000968567 |
| GO:BP | vasculature development | GO:0001944 | 0.001096359 |
| GO:BP | blood vessel morphogenesis | GO:0048514 | 0.002285541 |
| GO:BP | skeletal muscle satellite cell differentiation | GO:0014816 | 0.003260048 |
| GO:BP | anatomical structure formation involved in morphogenesis | GO:0048646 | 0.003497703 |
| GO:BP | tube development | GO:0035295 | 0.005311767 |
| GO:BP | ectodermal placode development | GO:0071696 | 0.006522326 |
| GO:BP | ectodermal placode formation | GO:0060788 | 0.006522326 |
| GO:BP | ectodermal placode morphogenesis | GO:0071697 | 0.006522326 |
| GO:BP | outflow tract morphogenesis | GO:0003151 | 0.015719732 |
| GO:BP | response to dsRNA | GO:0043331 | 0.016965756 |
| GO:BP | ventricular septum morphogenesis | GO:0060412 | 0.020271997 |
| GO:BP | cardiac septum morphogenesis | GO:0060411 | 0.025856381 |
| GO:BP | anatomical structure morphogenesis | GO:0009653 | 0.025914887 |
| GO:CC | cell-cell junction | GO:0005911 | 0.0127315 |
| KEGG | Cytokine-cytokine receptor interaction | KEGG:04060 | 0.003340364 |
| KEGG | Influenza A | KEGG:05164 | 0.003572826 |
| KEGG | Cytosolic DNA-sensing pathway | KEGG:04623 | 0.004027189 |
| KEGG | RIG-I-like receptor signaling pathway | KEGG:04622 | 0.006381957 |
| KEGG | Toll-like receptor signaling pathway | KEGG:04620 | 0.015546821 |
| KEGG | Necroptosis | KEGG:04217 | 0.034010343 |
| KEGG | NOD-like receptor signaling pathway | KEGG:04621 | 0.035120831 |
| KEGG | Herpes simplex virus 1 infection | KEGG:05168 | 0.045280228 |
| REAC | Regulation of IFNA signaling | REAC:R-GGA-912694 | 0.003259642 |
| REAC | Interferon Signaling | REAC:R-GGA-913531 | 0.003628535 |
| REAC | Interferon alpha/beta signaling | REAC:R-GGA-909733 | 0.004553912 |
| TF | Factor: IRF4; motif: MNNRAAANGAAA | TF:M08886 | 0.002054478 |
| TF | Factor: IRF-3; motif: NBNBTTTCSCTTT | TF:M01279 | 0.002501882 |
| TF | Factor: IRF-8; motif: AGTTTCW; match class: 1 | TF:M01665\_1 | 0.004639234 |
| TF | Factor: IRF; motif: NNGAAANTGAAANN; match class: 1 | TF:M08887\_1 | 0.015293909 |
| TF | Factor: NF-kappaB; motif: NGGGANTTYCCMNNNN | TF:M00774 | 0.018635864 |
| TF | Factor: IRF; motif: NNGAAANTGAAANN | TF:M08887 | 0.020701117 |
| TF | Factor: E2F-1; motif: TWSGCGCGAAAAYKR | TF:M00024 | 0.029447397 |
| TF | Factor: Sox-9; motif: RNACAAAGGVN; match class: 1 | TF:M01284\_1 | 0.04020694 |
GO:MF – Gene ontology molecular function
GO:BP – Gene ontology Biological process
GO:CC – Gene ontology cellular component
KEGG – KEGG pathway
REAC – Reactome
TF – Transcription factor
